# Factors associated with the co-utilization of oral rehydration solution and zinc for treating diarrhea among under-five children in 35 sub-saharan Africa countries: a generalized linear mixed effect modeling with robust error variance

**DOI:** 10.1186/s12889-024-18827-w

**Published:** 2024-05-16

**Authors:** Beminate Lemma Seifu, Bruck Tesfaye Legesse, Tirualem Zeleke Yehuala, Bizunesh Fantahun Kase, Zufan Alamrie Asmare, Getahun Fentaw Mulaw, Tsion Mulat Tebeje, Kusse Urmale Mare

**Affiliations:** 1 Department of Public Health, College of Medicine and Health Sciences, Samara University, Samara, Ethiopia; 2 Department of Pediatrics and Neonatal Nursing, School of Nursing and Midwifery, Institutes of Health Science, Wollega University, Nekemte, Ethiopia; 3 Department of Health Informatics, Institute of Public Health, University of Gondar, Gondar, Ethiopia; 4 Department of Public Health, College of Medicine and Health Sciences, Samara University, Samara, Ethiopia; 5 Department of Ophthalmology, School of Medicine and Health Science, Debre Tabor University, Debre Tabor, Ethiopia; 6 School of Public Health, College of Medicine and Health Sciences, Woldia University, Woldia, Ethiopia; 7 School of Pharmacy and Medical Sciences, Griffith University, Gold Coast, QLD 4222 Australia; 8 School of Public Health, College of Health Sciences and Medicine, Dilla University, Dilla, Ethiopia; 9 Department of Nursing, College of Medicine and Health Sciences, Samara University, Samara, Ethiopia

**Keywords:** Diarrhea, Multilevel analysis, ORS and zinc co-utilization, Sub-saharan Africa, Under-five children

## Abstract

**Introduction:**

Even though childhood diarrhea is treated with a simple treatment solution, it continues to be one of the leading causes of under-five child mortality and malnutrition globally. In resource-limited settings such as Sub-Saharan Africa (SSA), the combination of oral rehydration salts (ORS) and zinc is regarded as an effective treatment for diarrhea; however, its utilization is very low. The purpose of this study was to determine the proportion and associated factors of co-utilization of ORS and zinc among under-five children with diarrhea in SSA.

**Methods:**

The proportion and associated factors of co-utilization of ORS and zinc among under-five children with diarrhea in SSA were determined using secondary data analysis of recent Demographic and Health Surveys (DHS) of 35 SSA countries. The study included a total of 44,341 under-five children with diarrhea in weighted samples. A generalized linear mixed-effects model with robust error variance was used. For the variables included in the final model, adjusted prevalence ratios (aPR) with 95% confidence intervals (CI) were estimated. A model with the lowest deviance value were considered as the best-fitted model.

**Result:**

The pooled proportion of co-utilization of ORS and zinc for the treatment of diarrhea among under five children in SSA countries was 43.58% with a 95% CI (43.15%, 44.01%). Sex of the child, maternal age, residence, maternal educational and employment status, wealth index, media exposure, perceived distance to health facility and insurance coverage were statistically significant determinants of ORS and Zinc co-utilization for treating diarrhea among under five children in SSA.

**Conclusion:**

Only less than half of under-five children with diarrhea in SSA were treated with a combination of ORS and zinc. Thus, strengthening information dissemination through mass media, and community-level health education programs are important to scale up the utilization of the recommended combination treatment. Furthermore, increasing health insurance coverage, and establishing strategies to address the community with difficulty in accessing health facilities is also crucial in improving the use of the treatment.

## Background

Diarrhea is defined as the passage of three or more loose or watery stools in a day, which may differ from a child’s normal bowel movements in terms of frequency, consistency, or volume [1]. Childhood diarrhea, despite having a simple treatment solution, remains a leading cause of under-five mortality and malnutrition worldwide [2]. According to the 2017 report by the World Health Organization (WHO), diarrheal disease results in the deaths of approximately 525,000 children under the age of five each year [3], 60% of these deaths occur in 10 countries of Asia and Africa: Bangladesh, Democratic Republic of Congo, Ethiopia, India, Kenya, Niger, Nigeria, Pakistan, Tanzania, and Uganda. Diarrhoea, a condition associated with high-risk variables such as malnutrition, contaminated water sources, and a lack of access to treatment, remains a significant public health concern in low and middle-income countries (LMICs). Despite advances in medical science, the fatality rate associated with diarrhoea in LMICs remains high. The complex interplay of health, environmental, and socio-economic factors in these regions exacerbates the morbidity and mortality associated with diarrhoea, contributing to its continued prevalence [2].

To achieve the Sustainable Development Goal (SDG) of preventing preventable maternal and child deaths, it is imperative to prioritize the treatment of diarrhoea. Current global health statistics indicate that diarrhoea is a leading cause of death among children below the age of five, and it also has adverse effects on child’s health outcomes. As such, an unwavering focus on diarrhoea treatment is crucial in attaining the SDG targets [4]. Oral Rehydration Salts (ORS) and zinc are two cost-effective and highly efficacious treatments for diarrhoea. According to recent estimates, these treatments have the potential to avert up to 93% of diarrhoea-associated deaths. Consequently, the WHO and the United Nations Children’s Fund (UNICEF) have recommended the combined use of ORS and zinc to ensure optimal diarrhoea management [5, 6]. ORS is a crucial intervention in the management of diarrhea as it replenishes lost fluids and salts. Zinc supplementation, on the other hand, has been demonstrated to reduce the duration and severity of diarrhea episodes, thus mitigating the risk of recurrence in the short-term. Therefore, the complementary use of these two interventions can significantly improve the clinical outcomes of patients with diarrhea. Despite being a lifesaving treatment for diarrhea, the use of ORS remains critically low worldwide [2]. According to a 2017 report by UNICEF, less than half (44%) of the children under the age of five in LMICs received ORS, and fewer than 7% were treated with zinc. Unfortunately, many of these countries have high rates of mortality caused by diarrhea. By increasing access to these life-saving treatments, a significant number of deaths could be prevented [7].

Two studies conducted in Nepal and Kenya have revealed that a comparatively low proportion of primary caregivers administer zinc to manage diarrhea to their sick children. Specifically, the studies found that only 15% and 18% of primary caregivers in Nepal and Kenya, respectively, utilize zinc as a means of treating diarrhea [8, 9]. The management of diarrhea in accordance with the World Health Organization’s (WHO) guidelines, which includes the use of oral rehydration therapy, zinc, and continued feeding with increased frequency, is not widely practiced in Africa. The prevalence of this recommended approach remains low, ranging from 17% in Cote d’Ivoire to 38% in Niger [10]. In Ethiopia, 46% and 33% of under-five children with diarrhea received oral rehydration therapy and zinc respectively and only 17% received a combination of these [11].

A limited number of studies have identified factors that contribute to ORS and Zinc co-utilization among under-five children with diarrhea. These factors include maternal age, child age, child sex, maternal educational level, father’s educational level,  economic status, media exposure, residence, distance to health care facility and health insurance coverage [12–16].

Despite the benefits of using both ORS and zinc in treating diarrhea among children under of five, no studies have been conducted to determine their prevalence and associated factors in SSA. This study is the first of its kind to determine the extent and factors associated with the combined use of ORS and zinc in the region. The findings can serve as a baseline for government officials and policymakers to devise interventions aimed at improving the utilization of ORS and zinc.

## Methods

### Data source and study setting

This study was based on the most recent Demographic and Health Survey (DHS) data of 35 SSA countries. DHS is a nationally representative survey routinely conducted every five years and collects data on basic health indicators like mortality, morbidity, fertility, and maternal and child health-related characteristics. The survey used a two-stage stratified sampling technique to select the study participants. In the first stage, Enumeration Areas (EAs) were randomly selected based on the country’s recent population and using the housing census as a sampling frame, households were randomly selected in the second stage. Each country’s survey consists of different datasets including men, women, children, birth, and household datasets. For this study, the study population were mothers/ care givers of under five children, thus, we used the kids Record dataset (KR file).

### Source and study population

The source population of this study was all under-five children with diarrhea in SSA. The study population was all under-five children with diarrhea within the last two weeks preceding the survey. In the current study, a weighted sample of 44,341 children of under-five age were considered for final analysis.

Detailed information about DHS methodology can be found from the official database https://dhsprogram.com/Methodology/index.cfm.

### Study variables and definitions

#### Dependent variable

The co-utilization of ORS and zinc for the treatment of childhood diarrhea was dichotomized as “yes = 1” if the child uses both ORS and zinc for the treatment of childhood diarrhea and “no = 0” if the child does not use both ORS and zinc for the treatment of childhood diarrhea.

#### Independent variables

The independent variables were classified as community and individual-level variables. Sub-Saharan African region (East, West, Central, and South African regions), distance to the health facility and place of residence (urban or rural) were considered as community-level factors. Individual-level factors were maternal age, educational status of the mother, working status of the mother, number of under-five children, family size, wealth status, media exposure, sex of household head, health insurance, sex of the child, being twin, weight of the child at birth, birth order, and birth interval.

Media exposure: was created from three variables (frequency of listening to the radio, watching television, and reading newspapers or magazines). In this study, women who listened to radio or watched television or read newspaper/magazine at least once in a week were considered as having exposure to media (coded “Yes”) and otherwise labeled as not having media exposure (coded “No”).

### Data management and statistical analysis

Data extraction, coding, and analysis were done using Stata version 17 statistical software. The weighted data were used for analysis to restore the representativeness of the data. Since the DHS data has a hierarchical nature, the Intra-class Correlation Coefficient (ICC) was estimated to assess the clustering effect. The ICC indicated that there was a significant clustering effect (ICC *>* 10%). This study was a cross-sectional study and the prevalence of co-utilization of ORS and zinc was greater than 10%, and if we report the odds ratio, it could overestimate the association between co-utilization of ORS and zinc and the independent variables. In such cases, the prevalence ratio is the best measure of association, and therefore, multilevel Poisson regression analysis with robust variance was fitted to identify predictors of co-utilization of ORS and zinc among under five children. Variables with a p-value *<* 0.2 in the bi-variable multilevel Poisson regression analysis were considered for the multivariable analysis.

Four models were constructed for the multilevel Poisson regression analysis. The first model was a null model without explanatory variables to determine the extent of cluster variation in co-utilization of ORS and zinc. The second model was fitted with individual-level variables, the third with community-level variables, and the fourth with both individual and community-level variables at the same time. Because the models were nested, the deviance (-2Log-Likelihood Ratio (LLR)) was used to compare them, and the model with the lowest deviance was the best-fitted model for the data. Finally, the Adjusted Prevalence Ratio (APR) with its 95% confidence interval (CI) was reported, and variables with p value *<* 0.05 in the multivariable analysis were considered as significant predictors of co-utilization of ORS and zinc among under-five children.

### Ethical consideration

Permission to access the data used in this study was obtained from a measure demographic and health survey through an online request from http://www.dhsprogram.com. The data are publicly available from the program’s official database.

## Results

### Study participant characteristics

The present study incorporates a total weighted sample of 44,341 children under the age of five, who had diarrhea within the preceding two weeks prior to the survey. Out of all the children surveyed, 52.49% were male and 72.83% lived in rural areas. Additionally, 46.34% of the children were from poor households and 60.29% had mothers who did not receive formal education. A large majority of the children (94.95%) did not have health insurance coverage (Table 1).

**Table 1:** Socio-demographic and health related characteristics of study participants

Variable	Weighted Frequency (*N* = 41,492)	Percentage (%)
**Sex of the child**		
Male	21,781	52.49
Female	19,712	47.51
**Twin status**		
Yes	1,205	2.90
No	40,288	97.10
**Birth order**		
First	9,108	21.95
2–3	14,235	34.31
4–5	9,325	22.47
> 6	8,824	21.27
**Preceding birth interval (in months)**		
< 23	6,089	18.85
> 24	26,217	81.15
**Perceived child size at birth**		
Normal	18,169	43.79
Big	15,107	36.41
Small	8,216	19.80
**Age of the mother**		
15–19	3,203	7.72
20–24	10,627	25.61
25–29	11,203	27.00
30–34	8,124	19.58
35–39	5,361	12.92
40–44	2,305	5.56
45–49	669	1.61
**Maternal education**		
Have no formal education	16,478	39.71
Have formal education	25,015	60.29
**Maternal occupation**		
Employed	29,716	71.62
Not employed	11,776	28.38
**Marital status**		
Single	2,403	5.79
Married	36,104	87.01
Divorced / widowed/ separated	2,985	7.19
**Household wealth status**		
Poor	19,226	46.34
Middle	8,200	19.76
Rich	14,066	33.90
**covered by health insurance**		
Yes	1,942	5.05
No	36,531	94.95
Perceived difficulty to reach a health facility		
Big problem	16,625	42.50
Not a big problem	22,496	57.50
**Give ORS**		
Yes	16,422	39.58
No	25,070	60.42
**Give zinc**		
Yes	7,617	18.36
No	33,875	81.64
**Residence**		
Rural	30,218	72.83
Urban	11,274	27.17
**Sub-Saharan Africa region**		
Central Africa	8,881	21.40
Eastern Africa	7,745	18.67
Southern Africa	6,999	16.87
Western Africa	17,867	43.06

### Random effect and model comparison

The study assessed the presence of clustering effect using the Intraclass correlation coefficient (ICC), and model comparison was done using deviance. The ICC in the null model was 15.5%, indicating that about 15.5% of the total variation in Co-utilization of ORS and zinc was attributable to unmeasured or unmeasurable factors (random effects), and this variation was significant. Regarding model comparison, the final model (a model with lower deviance) was the best-fitted model (Table 2).

**Table 2:** Random effect and model comparison for factors associated with Co-utilization of ORS and Zinc

Parameter	Null model	Final model
ICC (%)	15.5 (12.4–30.7)	13.9 (10.7–26.4)
AIC	70,837.14	63,334.83
LL	-35,417.57	-31,638.41
Deviance	70,835.14	63,276.82

AIC Akaike Information criteria, LL Log likelihood

### Proportion and determinants co-utilization of ORS and zinc among under-five children with diarrhea

**Fig. 1:**
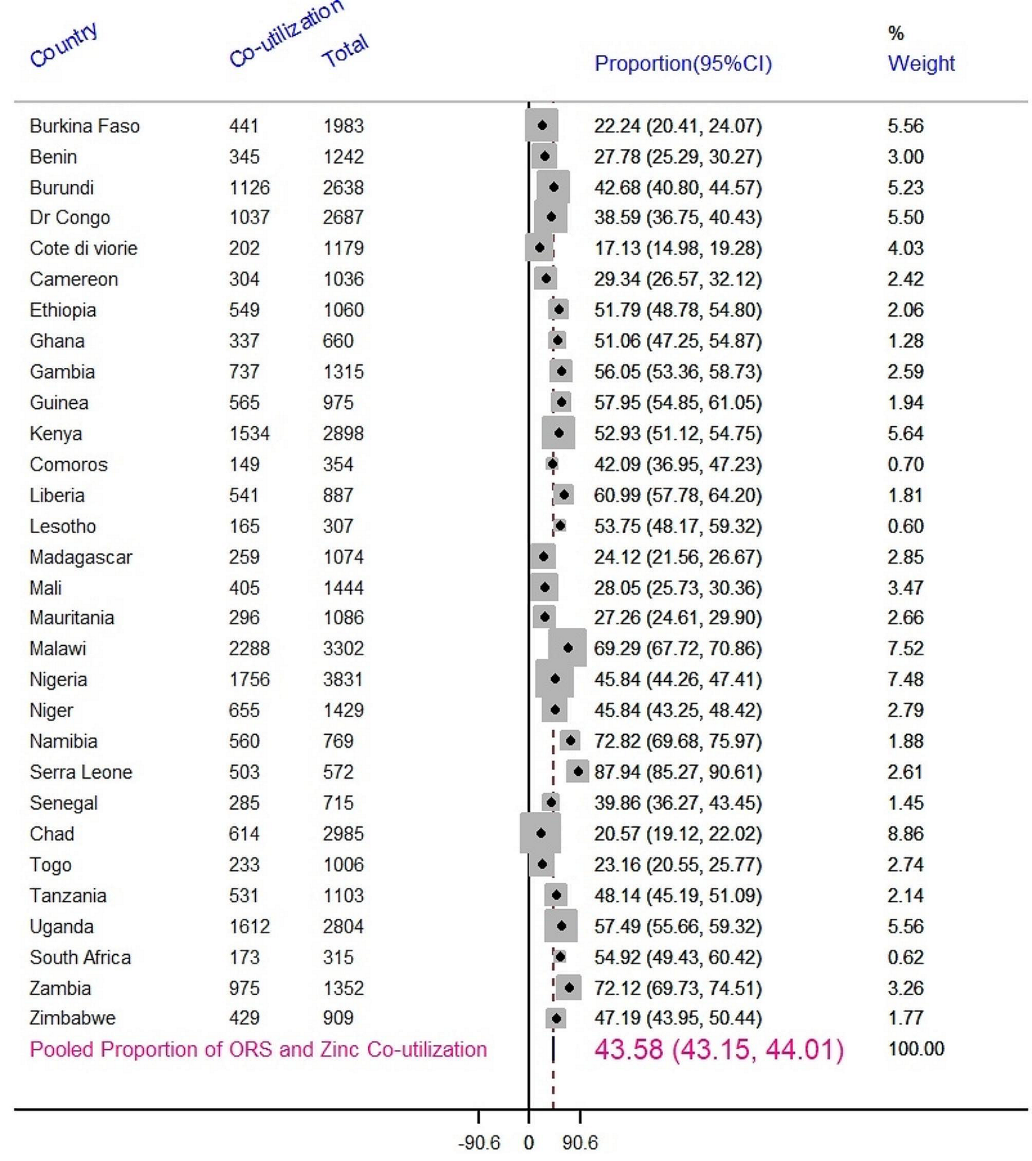
Forest plot of pooled proportion of co-utilization of ORS and zinc for the treatment of diarrhea among under five children in SSA countries

The pooled proportion of ORS and zinc co-utilization for the treatment of diarrhea among under five children in Sub-Saharan Africa was 43.58% with a 95% CI (43.15%, 44.01%). Cote di viorie had the lowest proportion of Co-utilization (17.3%; 95% CI (14.98%, 19.28%)) whereas Sierra Leone had the highest (87.94%; 95%CI (85.27%, 90.61%)) (Fig. 1).

In the final multivariable multilevel robust Poisson regression model; sex of the child, age and educational status of the mother, wealth status, media exposure, health insurance coverage were individual level variables while residence, distance to health facility and SSA region were community level variables found to be statistically significant (*P* ≤ 0.05) determinants of ORS and Zinc Co-utilization.

The proportion of ORS and Zinc Co-utilization for female children were 44.50% lower as compared to male children (aPR = 0.96, 95% CI; 0.94, 0.98). Mothers who reside in rural residencies were found to had a 4% (aPR = 0 0.96, 95% CI; 0.92, 0.99) decrease proportion of ORS and zinc co-utilization for the treatment of diarrhea. mothers whose age were between 15 and 19, 20–24 and 25–29 had 8%, 9% and 9% higher proportion of ORS and Zinc co-utilization for their children, respectively. Compared to children from non-educated mothers, those children born from mothers with formal education had 9% higher proportion of ORS and Zinc Co-utilization (aPR = 1.09, 95% CI; 1.06, 1.12). Similarly, children from middle and rich households had 45.89% and 49.07% higher proportion of ORS and Zinc Co-utilization respectively. Furthermore, children from households which has a health insurance coverage had 11% higher proportion of ORS and Zinc Co-utilization compared to their counterparts (aPR = 1.11, 95% CI; 1.05, 1.17) (Table 3). Mothers who had media exposure and who perceived distance to health facility is not a big problem had 8% (aPR = 1.08, 95% CI: 1.05, 1.11) and 7% (aPR = 1.07, 95%CI: 1.04, 1.10) higher proportion of co-utilization for the treatment of diarrhea for their under five children, respectively. Mothers who reside in eastern and southern Africa had 28% (aPR = 1.28, 95% CI: 1.22, 1.35) and 73% (aPR = 1.73, 95% CI: 1.64, 1.82) higher prevalence ORS and zinc co-utilization, respectively.

**Table 3:** Factors associated with co-utilization of ORS and Zinc for management of diarrhea among under-five children

Variable	Co-utilization of ORS and Zinc during Diarrhea Weighted frequency (%)		Prevalence Ratio (95% CI)	
	No	Yes	uPR	aPR
**Sex of the child**				
Male	12,648 (54.38)	10,612 (45.62)	1	1
Female	11,701 (55.50)	9,380 (44.50)	0 0.96 (0.94, 0 0.98)	0.96 (0 0.94, 0.98)*
**Age of the mother**				
15–19	1,974 (56.58)	1,515 (43.42)	1	1
20–24	6,073 (52.97)	5,391 (47.03)	1.07 (1.03, 1.13)	1.08 (1.03, 1.14)*
25–29	6,482 (53.73)	5,582 (46.27)	1.05 (1.00, 1.10)	1.09 (1.04, 1.15)*
30–34	4,693 (54.89)	3,857 (45.11)	1.03 (0 0.98, 1.08)	1.09 (1.03, 1.16)*
35–39	3,218 (56.88)	2,439 (43.12)	0 0.99 (0 0.93, 1.04)	1.06 (0.99, 1.13)
40–44	1,492 (61.61)	930 (38.39)	0 0.88 (0 0.82, 0 0.94)	0.98 (0.90, 1.06)
45–49	417 (60.05)	277 (39.95)	0 0.93 (0.84, 1.03)	1.06 (0 0.95, 1.18)
**Residence**				
Urban	6,055 (50.17)	6,015 (49.83)	1	1
Rural	18,293 (56.69)	13,978 (43.31)	0 0.89 (0 0.86, 0.91)	0 0.96 (0.92, 0.99)*
**Maternal education**				
Have no formal education	10,982 (63.19)	6,399 (36.81)	1	1
Have formal education	13,366 (49.58)	13,594 (50.42)	1.36 (1.33, 1.40)	1.09 (1.06, 1.12)*
**Maternal occupation**				
Not Employed	6,647 (56.45)	5,129 (43.55)	1	1
Employed	17,701 (54.36)	14,863 (45.64)	1.01 (0.98, 1.04)	1.04 (1.01, 1.07)*
**Household wealth status**				
Poor	12,104 (58.07)	8,739 (41.93)	1	1
Middle	4,700 (54.11)	3,985 (45.89)	1.08 (1.04, 1.11)	1.05 (1.01, 1.08)*
Rich	7,545 (50.93)	7,268 (49.07)	1.14 (1.11, 1.18)	1.04 (1.01, 1.07)*
**Birth order**				
First	5,176 (52.62)	4,660 (47.38)	1	1
2–3	8,025 (52.28)	7,325 (47.72)	0.99 (0 0.96, 1.02)	1.01 (0 0.97, 1.04)
4–5	5,559 (56.65)	4,255 (43.35)	0 0.92 (0 0.89, 0 0.95)	0 0.99 (0.95, 1.04)
> 6	5,589 (59.82)	3,753 (40.18)	0 0.85 (0.82, 0.88)	1.00 (0 0.94, 1.05)
**Media exposure**				
No	10,047 (60.44)	6,577 (39.56)	1	1
Yes	14,301 (51.60)	13,415 (48.40)	1.21 (1.18, 1.25)	1.08 (1.05, 1.11)*
**Covered by health insurance**				
No	20,438 (53.97)	17,432 (46.03)	1	1
Yes	966 (49.07)	1,003 (50.93)	1.10 (1.04, 1.15)	1.11 (1.05, 1.17)*
**Perceived difficulty to reach a health facility**				
Big problem	9,638 (56.35)	7,467 (43.65)	1	1
Not a big problem	12,179 (52.09)	11,202 (47.91)	1.10 (1.07, 1.13)	1.07 (1.04, 1.10)*
**Sub-Saharan Africa region**				
Central Africa	5,799 (65.19)	3,096 (34.81)	1	1
Eastern Africa	4,624 (50.09)	4,608 (49.91)	1.47 (1.40, 1.55)	1.28 (1.22, 1.35)*
Southern Africa	2,494 (34.56)	4,722 (65.44)	1.98 (1.89, 2.08)	1.73 (1.64, 1.82)*
Western Africa	11,431 (60.17)	7,566 (39.83)	1.17 (1.11, 1.23)	1.04 (0 0.99, 1.10)

^*^p ≤ 0.05, uPR: Unadjusted Prevalence Ratio, aPR: Adjusted Prevalence Ratio

## Discussion

According to the current study, the proportion of ORS and zinc co-utilization for the treatment of diarrhea among children under the age of five in SSA was 43.58% with considerable variation across countries. Sex of the child, maternal age, residence, maternal educational and employment status, wealth index, media exposure and insurance coverage, distance to health facility were statistically significant determinants of ORS and Zinc co-utilization for treating diarrhea among under five children in SSA.

In this study, compared to younger mothers, mothers aged 20 to 34 were more likely to utilize ORS and zinc for their diarrheal under-five children, this is in line with a study done in Nigeria [17]. This could be explained by life-history theory, which states that as the reproductive value decreases with age, investment in child health should increase [18]. It is a fact that older mothers are less likely to have more children [19]. Consequently, they tend to prioritize their current children’s health and seek appropriate treatment for their diarrheal child rather than future reproduction [20].

Compared to male children, female children were less likely to receive ORS and zinc combined treatment for diarrhea. This is supported by evidence indicating that girl children are at a disadvantage when it comes to therapeutic practices [20]. This could be because female bias is more prevalent in SSA nations, and as a result, parents are more inclined to seek medical treatment for their boy children than for their girl children [21].

Similar to study done in Sudan [22], Children in rural areas were less likely than those in urban areas to receive ORS treatment and zinc supplementation for the treatment of childhood diarrheal diseases among under 5 years. This might be due to rural residents having a relatively poor health care service access and do not have enough information about ORS and zinc in response to diarrhea as compared with urban residents. Due to less media exposure, rural residents may not be aware of ORS and zinc treatment, and may not seek medical attention as soon as their child develops diarrhea.

Mothers who had a formal education and formal employment were more likely to use ORS and zinc for diarrhea management compared to their counter parts. Studies done in Pakistan [23], Ethiopia [12, 24], Nigeria [13] and Nepal [25] report similar findings. This could be because women with formal employment are often more educated and educated women tend to have a good health-care seeking behavior for childhood illnesses including diarrhea [22]. In addition to this, those mothers are more likely to take the prescribed medications and have a better knowledge about the importance of co-utilization of ORS with Zinc for treating childhood diarrhea [13, 14, 26].

Similarly, households with a medium and rich wealth status were more likely to use ORS with zinc for their children who had diarrhea than households with a poor wealth status. This finding is consistent with the results of studies conducted in Ethiopia [15] and India [27]. This is because poor households in low and middle-income countries (LMICs), such as SSA countries, cannot afford the costs of healthcare services, including ORS and zinc. As a result, children from low-income families may not receive the appropriate treatment when they have diarrhea [28].

In line with a study done in India [29], media exposure were associated with increased co-utilization of ORS and zinc to treat diarrhea in children under five. It is worth noting that access to mass media cannot be equated with exposure to co-utilization messages, as it may be a proxy for other variables such as education. Nonetheless, messages pertaining to health promotion and management of childhood diarrhea can significantly enhance the knowledge of mothers or caregivers on appropriate management of childhood diarrhea [25].

Mothers who perceived distance to health facility is not a problem found to use ORS and Zinc for their diarrheal child. This could be because mothers who have difficulty getting to a health facility must utilize transportation or travel a great distance to get there, which adds to their reasons for not seeking health care for their children [30]. Furthermore, mothers who live near a healthcare facility are more likely to visit the institution since they do not have to pay for transportation [31, 32].

When compared to mothers who are not members of health insurance, mothers who are members of health insurance are more likely to use ORS and Zinc for their children who have diarrhea. This could be because households that do not have health insurance and do not have health facilities nearby may find it difficult to access health care services.

### Strength and limitation

The study’s strength is that it is based on nationally representative data and uses appropriate analysis techniques. This is also the first study to look at the factors that influence ORS and zinc co-utilization in children under the age of five in SSA. Despite the aforementioned strengths, the measure of zinc utilization practice was based on mother’s recall, which could lead to recall bias. Second, due to the cross-sectional nature of the data, a clear temporality (cause and effect relationship) between the dependent and independent variables was not established. Moreover, the data provided by the DHS is insufficient to assess the effectiveness of the co-utilization scale-up. This is mainly due to the lack of critical information needed to evaluate the public and private sector scale-up strategies, accessibility of co-packed ORS and zinc, over-the-counter availability, household expenses, health-seeking behaviors, and marketing strategies employed.

## Conclusion

In this study older maternal age, having formal education, middle and rich wealth status, media exposure, covered by health insurance and distance to health facility were predisposing factors for ORS and Zinc Co-utilization. While the mere access to mass media cannot be equated with exposure to co-utilization messages, it can serve as a viable proxy for other determinants, such as education. It is thus recommended to enhance media coverage for the promotion of health and the management of childhood diarrhea. Furthermore, increasing maternal education and improving economic status is recommended to have an increased ORS and zinc Co-utilization rate among under-five children with diarrhea in the region. Furthermore, through universal maternal health insurance, emphasis should be placed on policy and intervention programs aimed at ending preventable deaths of children under the age of five and ensuring universal access to quality healthcare services in SSA. This is critical because the distance and cost barriers to seeking adequate healthcare services may be difficult for mothers, increasing the likelihood of a child’s death.

## Data Availability

All result-based data are in the manuscript. In addition, the dataset can be accessed from the measure DHS Program through https://www.dhsprogram.com.

## Abbreviations

aPR: Adjusted Prevalence Ratio
CI: Confidence Interval
uPR: Unadjusted Prevalence Ratio
DHS: Demographic and Health Survey
ICC: Intraclass Correlation Coefficient
LL: Log likelihood
UNICEF: United Nations Children’s Fund
